# Computer-Vision-Based Vibration Tracking Using a Digital Camera: A Sparse-Optical-Flow-Based Target Tracking Method

**DOI:** 10.3390/s22186869

**Published:** 2022-09-11

**Authors:** Guang-Yu Nie, Saran Srikanth Bodda, Harleen Kaur Sandhu, Kevin Han, Abhinav Gupta

**Affiliations:** Department of Civil, Construction, and Environmental Engineering, North Carolina State University, Raleigh, NC 27695, USA

**Keywords:** computer vision, acceleration response, target tracking, sparse optical flow

## Abstract

Computer-vision-based target tracking is a technology applied to a wide range of research areas, including structural vibration monitoring. However, current target tracking methods suffer from noise in digital image processing. In this paper, a new target tracking method based on the sparse optical flow technique is introduced for improving the accuracy in tracking the target, especially when the target has a large displacement. The proposed method utilizes the Oriented FAST and Rotated BRIEF (ORB) technique which is based on FAST (Features from Accelerated Segment Test), a feature detector, and BRIEF (Binary Robust Independent Elementary Features), a binary descriptor. ORB maintains a variety of keypoints and combines the multi-level strategy with an optical flow algorithm to search the keypoints with a large motion vector for tracking. Then, an outlier removal method based on Hamming distance and interquartile range (IQR) score is introduced to minimize the error. The proposed target tracking method is verified through a lab experiment—a three-story shear building structure subjected to various harmonic excitations. It is compared with existing sparse-optical-flow-based target tracking methods and target tracking methods based on three other types of techniques, i.e., feature matching, dense optical flow, and template matching. The results show that the performance of target tracking is greatly improved through the use of a multi-level strategy and the proposed outlier removal method. The proposed sparse-optical-flow-based target tracking method achieves the best accuracy compared to other existing target tracking methods.

## 1. Introduction

Computer vision techniques have led to great advancements in detecting and tracking objects and are being increasingly researched for applications in vibration monitoring of structural systems to replace conventional contact-based discrete sensors [1,2,3,4,5,6]. In computer-vision-based vibration monitoring methods, the displacement time history of a specific target on the structure is measured by the tracking changes in the video frames, and then the displacement response is converted to acceleration response using numerical differentiation methods. Compared with conventional measurement, visual-sensing-based methods do not require the installation and maintenance of expensive sensor setups. Region-based target tracking approaches often employ a *predefined template* such as a physical template and region of interest (ROI) for vibration monitoring. However, these techniques require installment of targets, which makes the process tedious [1,3]. Moreover, predefined templates are easily occluded by adverse factors such as partial occlusion, shape deformation, scale change, and rotation, which are challenges for visual tracking.

*Keypoint* is another kind of target for structural monitoring, in which a point on the structure that stands out from the rest is used, such as the corner point or ending point of a line segment. Many studies [7,8] utilize a feature-matching-based target tracking algorithm to track the motion of a set of keypoints. Feature matching can be easily affected by changes in illumination, noise, and motion blurring. These disadvantages are critical issues for field applications and thus have limited the adoption of vision-based monitoring methods. The robustness of the keypoints tracking can be improved by using a sparse optical flow algorithm because it considers constraints on the flow field smoothness and the brightness constancy [9,10]. Due to the brightness constancy constraint, the values of image brightness across all images are restricted. Therefore, optical-flow-based keypoint tracking is immune to the changes in brightness compared to feature-matching-based keypoint tracking. Most researchers combine the Lucas–Kanade (LK) algorithm [11] with different feature detectors to track keypoints. A few of these studies employ an outlier removal method to improve the tracking performance, but the technique based on sparse optical flow is not fully explored. Specifically, existing sparse-optical-flow-based vibration monitoring methods do not perform very well when calculating the vibration of structures with large displacements. Therefore, it is useful and important to obtain multi-point movement records and analyze them for a comprehensive assessment of structural response.

In this study, a novel sparse-optical-flow-based target tracking approach for structural vibration monitoring is proposed, where the conventional sparse optical flow algorithm (i.e., LK) is enhanced to track a set of sparse keypoints accurately. A multi-level strategy is applied to the LK algorithm to enhance the large motion vector calculation. Moreover, Oriented Fast and Rotated Brief (ORB), a corner extraction algorithm, is used to detect the keypoints, and an outlier removal method based on Hamming distance and interquartile range (IQR) score is introduced to minimize the error between the experimental response versus the vision-based response. The accuracy of the proposed method is evaluated by measuring the acceleration response from a three-story shear building in the laboratory subjected to three different harmonic transient excitations. The results from the proposed method are also compared with those from the recent existing target tracking methods that are based on different techniques such as sparse optical flow, feature matching, dense optical flow, and template matching.

The manuscript is divided into eight sections: Section 2 describes the existing studies related to target tracking methods. The proposed method is presented in Section 3. Section 4 introduces the vision-based sensing system used for experimental vibration tests. The description of structural laboratory experiment is presented in Section 5. Section 6 presents the qualitative and quantitative assessment of the proposed method and a comparison with various vision-based target tracking methods. The discussion of results is presented in Section 7. Finally, the conclusions of this research are presented in Section 8.

## 2. Target Tracking Methods: Background Literature

This section reviews various target tracking methods for vision-based vibration monitoring that are based on four techniques: sparse optical flow, feature matching, dense optical flow, and template matching.

### 2.1. Sparse Optical Flow

In such target tracking methods, a set of keypoints are first extracted in the current frame, and then the optical flow vectors are calculated to track the locations of keypoints in the next frame. This technique mainly contains three parts, i.e., keypoints detection, optical flow estimation, and outlier removal [12]. The LK algorithm [11] is the most popular algorithm used for optical flow estimation, but it is limited to tracking targets that have large motion between two consecutive frames. The most prevalent keypoints are extracted by the Harris corner detector [9,13], Shi–Tomasi corner detector [10,14], scale-invariant feature transform (SIFT) algorithm [13], and speeded up robust features (SURF) algorithm [15,16]. However, not all sparse-optical-flow-based target tracking methods used for structural vibration monitoring implement outlier removal methods. Maximum Likelihood Estimator SAmple Consensus (MLESAC) modeling fitting [16,17] and bidirectional error detection [18] are two methods that are used to eliminate the outliers of tracked keypoints. However, the MLESAC-based outlier removal method does not consider the direction of outliers, and the bidirectional error detection-based outlier removal method does not consider eliminating the slight motion of background and other non-rigid objects.

### 2.2. Feature Matching

Such target tracking methods firstly detect a set of keypoints in each of the two frames, and then employ feature matching to search for the best-matched keypoint pairs from the two frames [12]. This mainly consists of four steps, i.e., keypoints detection, feature description, keypoints matching, and outlier removal. Existing feature-matching-based target tracking methods employ various algorithms for each step. The keypoint detector includes circular Hough transform (CHT) [19], scale-invariant feature transform (SIFT) [7,8,20], response matrix [21], and ORB [22]. The feature descriptor includes SIFT [7], Fast Retina Keypoint (FREAK) [21], and Visual Geometry Group (VGG) [8,20]. The minimum Euclidean distance [7] and Hamming distance [21] are often used for searching the initial keypoints matching between different frames. After initial matching, coherent point drift (CPD) algorithm [19], trimmed mean algorithm [7], least squares fit algorithm [21], and RANdom SAmple Consensus (RANSAC) [20] are used for outlier removal.

### 2.3. Dense Optical Flow

Such target tracking methods calculate the motion vector of every pixel within the predefined template [13,23]. Compared to sparse keypoints-based techniques, this kind of technique does not need other steps to remove the outliers. Existing methods employ different kinds of dense optical flow algorithms. For example, Khaloo et al. [13] estimated the dense optical flow using four methods, i.e., LK [11], Horn–Schunck (HS) [24], Black and Anandan (BA) [25], and classic+NL (CLNL) [26]. In the study of Celik et al. [23], existing dense flow methods [27,28,29] were utilized to track larger crowds. Won et al. [30] used Deepmatching [31] and Deepflow [32] to find dense correspondence between two image frames. Dong et al. [1] compared the optical flow results generated by six methods, i.e., HS, LK with pyramid and sparse to dense interpolation (LKPyrSD), BA, Farneback [29], CLNL, and FlowNet2 [33].

### 2.4. Template Matching

Such target tracking methods detect a predefined template in a reference frame and then search for the area in a new frame that is most correlated to the predefined template. This technique is easy to implement without user intervention and has been validated to work well for vibration monitoring. The predefined templates used for structural vibration monitoring mainly consist of two types: natural templates and artificial templates. For example, an ROI [22] and segmented screws [34] are used as natural targets. Compared to natural targets, many studies predefine artificial targets as the templates, such as concentric rings with a gradual blend from black to white at the edges [35,36,37], ArUco markers [3,9,38], coded and uncoded optical target arrays [39,40], circular border pattern with line pattern including multiple intersected lines [41,42,43,44], artificial quasi-interferogram fringe pattern (QIFP) [45], speckle pattern [46], illuminated light source [47], and retro reflective materials [48].

## 3. Proposed Method

**Figure 1:**
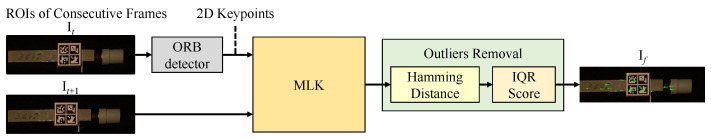
Flowchart of the proposed sparse-optical-flow-based target tracking method. ORB: Oriented Fast and Rotated Brief, MLK: multi-level Lucas–Kanade algorithm, IQR: interquartile range.

This section introduces the proposed sparse-optical-flow-based target tracking method used for structural vibration monitoring. Compared to the existing studies [9,15], a new combination is created by employing different methods for keypoints extraction and removal of outliers and applying a multi-level strategy on the LK algorithm to enhance the target tracking. As shown in Figure 1, this combination takes the ROIs cropped from two consecutive frames ($I_{t}$, $I_{t+1}$) as input, and outputs the motion trajectory (green lines) for each sparse keypoint (green dots) on the previous frame, $I_{t}$. Keypoints are first extracted in $I_{t}$, and then optical flow vectors ($I_{f}$) are calculated to track the locations of keypoints in the next (i.e., current) frame, $I_{t+1}$. The key elements in the proposed method are described below.

**ROI** indicates the location that is being monitored on the vibrating structure (i.e., girder) for keypoints tracking. As the girder is a rigid structure, the displacement of all keypoints will be the same. Therefore, ROI is defined manually by drawing a box on the area with rich features in the initial frame of the video, for example, all of the pixels that correspond to the right part of the top floor (see Figure 1). Then the first image and the successive images captured by the ROI are tracked continuously.

**ORB** is widely used in computer vision tasks such as object detection and stereo matching [49,50]. It is basically a fusion of Features from Accelerated Segment Test (FAST) keypoint detector [51] and Binary Robust Independent Elementary Features (BRIEF) descriptor [52] with many modifications to enhance the performance. ORB performs as well as SIFT in the task of feature detection and it is better than SURF [49]. In this paper, ORB is employed to detect two-dimensional (2D) keypoints in the ROIs of $I_{t}$.

**LK Algorithm** is a widely used method for motion vector estimation, which is based on the assumption of brightness constancy [11]. Consider a pixel I(x,y,t) in the reference frame, and it moves by a distance of (Δx,Δy) in the next frame, which is taken after a period of time Δt. Assuming that the pixels are the same and their intensity does not change over time, one can write:

(1) I(x,y,t)=I(x+Δx,y+Δy,t+Δt)

For two continuous frames $I_{t}$ and $I_{t+1}$, a small w×w window is considered in the neighborhood of a keypoint (x,y) in $I_{t}$, and a matched pixel (x+Δx,y+Δy) in $I_{t+1}$ is located by using a Gauss–Newton algorithm, where the target function shown in Equation (2) is minimized.

(2) $$\min_{\Delta x,\;\Delta y}{\left\|I_{t}\;\left(x,\;y\right)-I_{t+1}\;\left(x+\Delta x,\;y+\Delta y\right)\right\|}^{2}$$

**Figure 2:**
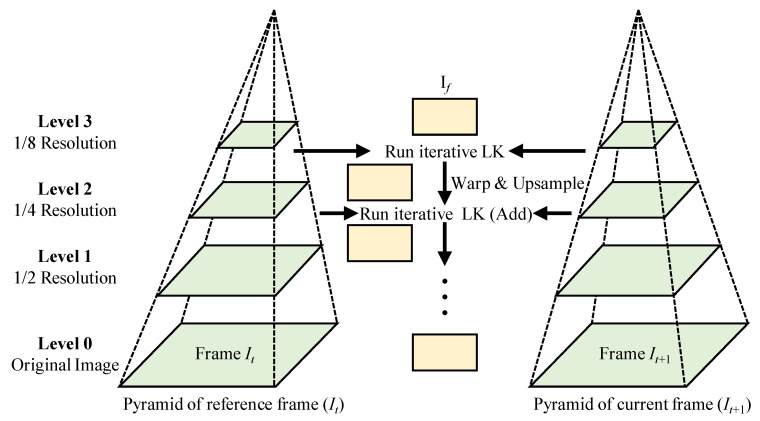
Flowchart of multi-level optical flow strategy that is used in the proposed method.

**Multi-level Optical Flow Strategy** allows the flow field to be estimated at coarser levels and then be fine-tuned by increasing the resolution of images. Adelson et al. [53] investigated the use of the pyramid approach to develop a multi-level optical flow strategy. As shown in Figure 2, the Gaussian pyramid is employed and the resolution of the image is reduced at each level while climbing the pyramid. To develop a multi-level strategy, the *number of levels* needs to be specified, which is one of the critical parameters used in the image pyramid. A finer level leads to greater accuracy of the algorithm, but it would also lead to a higher cost of computational resources. Another important parameter that needs to be specified is the *scaling factor*, which determines the extent of downsampling images in the pyramid. As shown in Figure 2, the optical flow is estimated based on a multi-level optical flow strategy with its subsequent warping steps, where a four-level image pyramid is first created for each frame by downsampling the image with the scaling factor of n=0.5. Then, the optical flow is computed at lower-resolution images, which serves as the initialization for higher-resolution pyramid levels. Due to the brightness constancy assumption, the LK algorithm can only estimate small displacements. Therefore, in this study, the multi-level strategy is combined with the LK algorithm to manage large displacements [23].

In this study, the proposed **Outlier removal** method is based on Hamming distance and IQR score. This is conducted after obtaining the initially matched keypoints from the MLK algorithm to improve the accuracy of tracking and eliminate the outliers of tracked keypoints in the next frame. The similarity between all the initially matched pairs of keypoints is checked by calculating the **Hamming distance**, $d(a_{t},a_{t+1})$, based on the ORB descriptors (i.e., $a_{t}$ and $a_{t+1}$) of the matched keypoints. The keypoints are eliminated as outliers if the Hamming distance is greater than the maximum of either $2d_{min}$ or a threshold value *s*.

(3) $$d\left(a_{t},a_{t+1}\right)\geq max\left(2\times d_{min},\;s\right)$$

where $d_{min}$ is the minimum Hamming distance of all initially matched pairs. The threshold value *s* is chosen based on expert judgment.

Subsequently, the keypoints are removed as outliers based on the IQR value. The displacement decrements of each matched keypoint in Euclidean space, horizontal direction, and vertical direction are calculated. The selected matched keypoints are sorted in the order from least to greatest based on these three displacements, respectively. Then, the **IQR value** of each of the three sorted displacements is calculated using Equation (4):

(4) $$IQR=Q_{3}-Q_{1}$$

where $Q_{1}$ and $Q_{3}$ are the first and third quartiles of each kind of sorted displacements. For each pair of matched keypoints, if any one of its three kinds of relative displacements lies outside a specified range $\left[Q_{1}-r\times IQR,Q_{2}+r\times IQR\right]$, the keypoint is regarded as an outlier and is removed. In this study, r=0.8 is selected based on a qualitative study, which is a trade-off between the accuracy of keypoint tracking and the number of final matched keypoints; however, the results are not presented here for brevity.

Finally, the **displacement decrement** of the monitored vibrating structure between each of the two consecutive frames is calculated by averaging the decrements for each keypoint following the previous studies [18].

## 4. Vision-Based Sensing System

**Figure 3:**
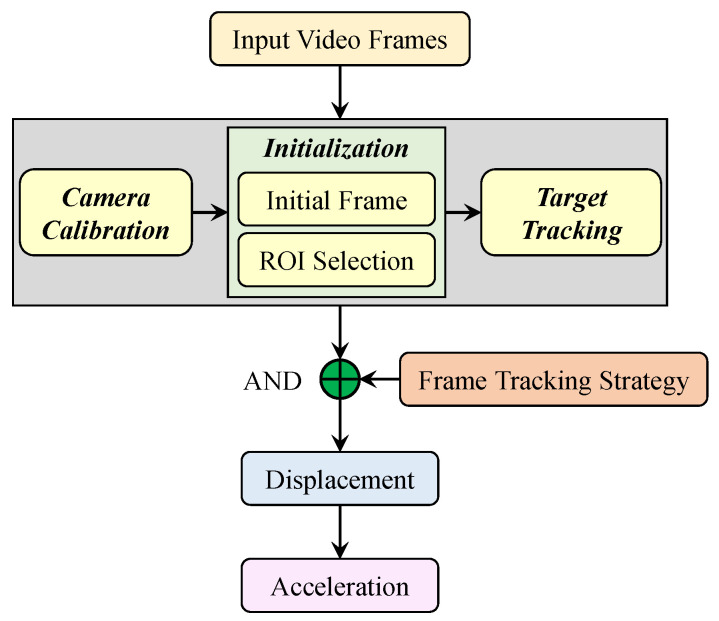
Flowchart of the visual sensing system.

In this study, the visual sensing system used for structural vibration monitoring is based on target tracking techniques. As shown in Figure 3, this system takes the video frames that record the vibration of a structure as an input and outputs the acceleration time histories of the structural vibration. It consists of two components: (i) camera calibration and scale conversion; and (ii) frame tracking strategies and displacement calculation. To save on computational resources, an ROI is defined in the first frame.

### 4.1. Camera Calibration and Scale Conversion

In this study, image distortion removal and scale conversion to calibrate the camera are implemented. An offline camera calibration method, Open Source Computer Vision Library (OpenCV), is used to remove the video image distortion [54]. The radial distortion and tangential distortion are two major kinds of distortions in pinhole cameras. The lens distortion is corrected by accounting for the radial distortion and the tangential distortion according to Equation (5).

(5) $$\begin{array}{c} x_{\mathrm{distorted}}=x+x\left(1+k_{1}r^{2}+k_{2}r^{4}+k_{3}r^{6}\right)+\left(2p_{1}xy+p_{2}\left(r^{2}+2x^{2}\right)\right)\\ y_{\mathrm{distorted}}=x+y\left(1+k_{1}r^{2}+k_{2}r^{4}+k_{3}r^{6}\right)+\left(p_{1}\left(r^{2}+2y^{2}\right)+2p_{2}xy\right) \end{array}$$

where (x,y) is the undistorted pixels, and $r^{2}=x^{2}+y^{2}$. The terms $k_{1}$, $k_{2}$, and $k_{3}$ represent the radial distortion coefficients, while $p_{1}$ and $p_{2}$ are the tangential distortion coefficients. The camera-specific distortion coefficient values used in this study are presented in Section 5 of this manuscript.

After correcting for lens distortion, a scale ratio *s* is used to convert the image coordinates (i.e., pixels) to actual spatial coordinates (e.g., millimeters), and it is given by Equation (6).

(6) s=d/D

where, *d* is the distance between two points of an object (e.g., chessboard) in the actual spatial coordinate, while *D* is its corresponding distance in the image coordinate.

### 4.2. Frame Tracking Strategies and Displacement Calculation

**Figure 4:**
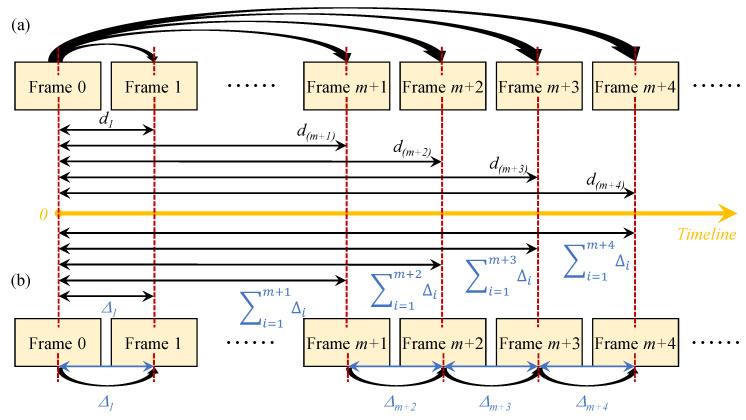
Two frame tracking strategies: (**a**) fixed-frame strategy, (**b**) updated-frame strategy; *d*: absolute displacement, Δ: relative displacement.

The displacement time history is often calculated by either employing a fixed-frame strategy or an updated-frame strategy [1,55]. The main difference between these two strategies is whether the *reference frame* is kept fixed or is updated when calculating the displacement for each tracked target. Figure 4a shows the **fixed-frame** strategy, where the first frame (i.e., Frame 0) is always used as the reference frame. The absolute displacement of each target at every single time instant is calculated by subtracting the location coordinate of the target in Frame 0 from the location coordinate in the current frame (e.g., Frame *m* + 1, Frame *m* + 2, Frame *m* + 3). Figure 4b shows the **updated-frame** strategy. The displacement decrement $\Delta_{i}$ between two consecutive frames (e.g., Frame *m*+1 and Frame *m* + 2, Frame *m* + 2 and Frame *m* + 3) is calculated, and the absolute displacement at every instant of time is the accumulation of all previous $\Delta_{i}$. Then, the actual displacements are obtained by multiplying the displacements in pixel coordinates with the calculated scale ratio, *s*. Finally, the proposed sparse-optical-flow-based target tracking approach is combined with the updated-frame strategy to calculate the acceleration time history.

## 5. Experimental Setup for Measurement

**Figure 5:**
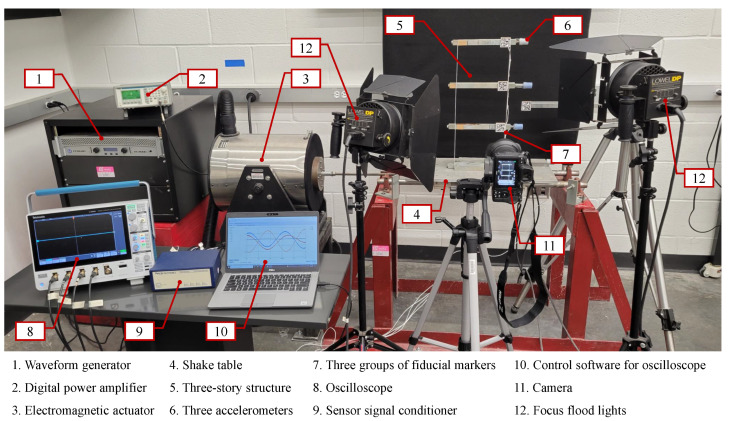
Overview of the experimental setup.

This section describes the experimental setup and the different systems used to evaluate the performance of the target tracking approach in structural vibration monitoring. The overview of the experimental setup is shown in Figure 5. A *three-story shear building structure* is fixed on the shake table and subjected to harmonic loads excitation using an *excitation system*. A *reference system* measures the acceleration time series response for the vibration of each floor under different excitation frequencies. A *vision sensor system* records the structural vibration for acceleration calculation. The technical specifications of instruments used in each system are tabulated and included in the Supplementary Document (Tables S1 and S2).

### 5.1. Experimental Three-Story Shear Building Structure

**Figure 6:**
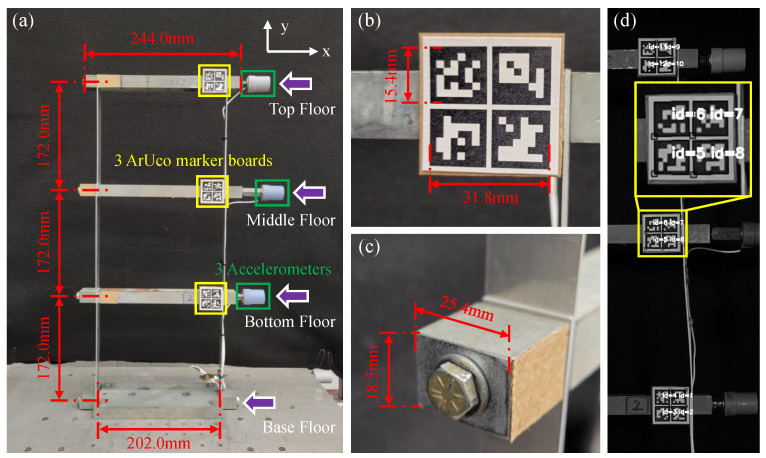
Experimental three-story shear building structure. (**a**) Overview of the structure; (**b**) close-up shot of the ArUco marker board fixed on the top floor; (**c**) left end of the lumped mass steel stick of the middle floor; (**d**) detected ids of the fiducial markers.

It consists of two aluminum columns and one lumped mass steel girder on each floor with its base fixed rigidly to a uniaxial shake table as shown in Figure 6a. The specifications of the structure are as follows: height of each floor H=172.0 mm, size of each floor is $w_{1}\times h_{1}\times l_{1}=$ 25.4 mm × 18.5 mm × 244.0 mm, mass of each floor $m_{1}=$ 0.914 kg, size of each column $w_{2}\times h_{2}\times l_{2}=$ 1.5 mm × 25.2 mm × 194.0 mm, mass of each column $m_{2}=$ 0.020 kg, distance between each pair of columns W= 202.0 mm. The three natural frequencies of the structure are 3.84 Hz, 10.96 Hz, and 15.61 Hz.

### 5.2. Excitation System

It consists of the waveform generator, digital power amplifier, electromagnetic actuator, and shake table. In this study, harmonic base excitations are simulated in the horizontal direction at three frequency levels: 2 Hz, 5 Hz, and 10 Hz. These frequencies are chosen because most structures have fundamental frequencies in the range of 2–10 Hz. In addition, for safety against the in-house operational vibrations at industrial facilities such as the pump-induced vibrations, the frequencies are typically on the order of 5–10 Hz [56]. For each excitation frequency level, the time of excitation is chosen as 20 s, 10 s, and 10 s, respectively.

In industrial facilities and nuclear power plants, the vibrations that occur during in-plant operations, such as pump-induced vibrations, flow-assisted vibrations, or seismic vibrations, are transient in nature with noise rather than steady state. Therefore, in the experimental setup, the excitation frequency is kept fixed but the amplitude of vibration is changed continuously during the structure’s excitation to capture the transient nature of measurements in reality.

### 5.3. Reference System

In this study, three uniaxial high-sensitivity piezoelectric accelerometers (PCB 308B02) with a sensitivity of 1000 mV/g and frequency range of 250–3000 Hz (±10%) are mounted on each floor of the shear building (see Figure 6) to capture the structural acceleration versus time responses in the horizontal direction. As shown in Figure 5, a sensor signal conditioner is employed to convert the electrical signal captured from accelerometers into the type of signal that is read by the oscilloscope. Then, an oscilloscope is utilized to display, store, and transfer the waveform data as .csv files.

### 5.4. Vision Sensor System

It consists of two parts: data acquisition and data processing.

#### 5.4.1. Data Acquisition

In this study, a Nikon Z 7 Mirrorless Digital Camera equipped with a Nikon NIKKOR Z 24–70 mm f/4 S Lens is positioned at a distance of 900 mm away from the frame to record the structural vibration in the video, as shown in Figure 5. The values of camera lens distortion coefficients are $k_{1}=0.00224349$, $k_{2}=-0.15135992$, $k_{3}=0.37956948$, $p_{1}=0.00679483$, and $p_{2}=-0.00144892$. The calculated scale ratios of the videos corresponding to the structural vibration under three excitation frequencies (2 Hz, 5 Hz, and 10 Hz) are 0.39596, 0.38319, and 0.38435, respectively. The details of the video image distortion removal for the vision-based sensing system are shown in Section S2 of the Supplementary Document.

The angle of the lens is an important factor and impacts the results. For instance, the accuracy of the algorithm diminishes with the increased camera angle [3,21], but the monitoring angles of less than 15 degrees do not have a detrimental effect on system performance [16]. This research focuses on evaluating the accuracy of target tracking methods in measuring the acceleration of the structural vibration. Hence, the optical axis of the lens is oriented perpendicular to the motion axis (i.e., facing straight on the side of the frame) to eliminate the impact of the angle of the lens. However, this work can be extended for out-of-plane vibrations. For instance, the combination of multiple cameras to estimate the movement in three directions (i.e., *x*, *y*, and *z*) is similar to digital coordination but from different views and using targets (i.e., QR codes/fiduciary markers). Lastly, checking the normal configuration of the camera can be a simple visual check, making sure that the video is not blurry. This is because the accuracy of the system is dictated by the accuracy of target tracking. The accuracy of target tracking depends on the accuracy of target detection, which depends on the image quality (mainly blurriness).

To eliminate the motion blur when recording the fast-moving structure, the frame rate is set to 120 fps (frames per second) and the resolution is set to 1280 px × 960 px. Moreover, a moving object in the video will be blurred if the shutter speed of the camera is not fast enough. A 1/8000 s exposure can remove motion blur for almost any image, but fast shutter speeds will lead to dark images. To solve this issue, two Lowel DP focus flood lights (120–240 VAC) are placed in front of the vibrating structure as compensation to obtain a bright image.

#### 5.4.2. Data Processing

Data processing with help of a graphics processing unit (GPU) can make the algorithm work fast. In this research, a widely used process is employed in which the data are received on the CPU and then transmitted to GPU for further processing. It is important to note that this has no impact on the buffer size. To compute the acceleration, a Dell Alienware Aurora R7 desktop with 8th Gen Intel Core i7-8700 and NVIDIA GeForce GTX 2080 GPU is employed.

## 6. Qualitative and Quantitative Assessment of Proposed Method

In this section, the proposed vision-based target tracking method for structural vibration monitoring is evaluated qualitatively and quantitatively through the laboratory-scale experiment. The proposed method is also compared with various existing target tracking methods in the literature.

### 6.1. Implementation of the Proposed Method

**Figure 7:**
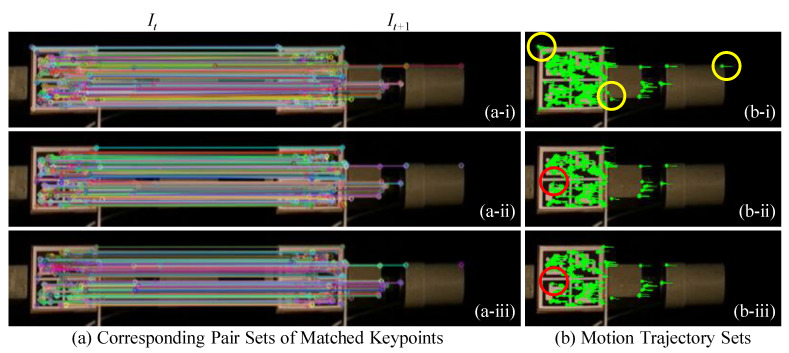
Qualitative example of the sparse-optical-flow-based target tracking. Pairs of matched keypoints in the ROIs of the previous frame, $I_{t}$, and the current frame, $I_{t+1}$, after implementing (**a-i**) sparse optical flow calculation, (**a-ii**) Hamming distance-based outlier removal method, and (**a-iii**) IQR score-based outlier removal method. Motion trajectories for the matched keypoints in the ROI of $I_{t}$ from time *t* to t+1, after implementing (**b-i**) sparse optical flow calculation, (**b-ii**) Hamming distance-based outlier removal method, and (**b-iii**) IQR score-based outlier removal method.

The proposed target tracking method is implemented in C++ programming language by carrying out three key steps: (i) sparse optical flow calculation, (ii) Hamming distance-based outlier removal method, and (iii) IQR score-based outlier removal method. Figure 7 shows two instances of frame $\left(I_{t},I_{t+1}\right)$ to qualitatively compare the effects of three steps. The colored circles in Figure 7 represent the keypoints 2D position: $\left(x_{i},y_{i}\right)$ detected by the ORB detector in frame $I_{t}$ and the optical flow algorithm in frame $I_{t+1}$, and each of them is a local extremum whose pixel intensity is greater or smaller than all its neighbors. Images in Figure 7a show the pairs of matched keypoints in the ROIs of the previous frame, $I_{t}$, and the current frame, $I_{t+1}$, after implementing all three steps. The colored lines connect the matched keypoints in the ROI of $I_{t}$ and their corresponding keypoints in $I_{t+1}$. Images in Figure 7b show the motion trajectories (green lines) for the matched keypoints (greens dots) in the ROI of $I_{t}$ from time *t* to t+1.

**Figure 8:**
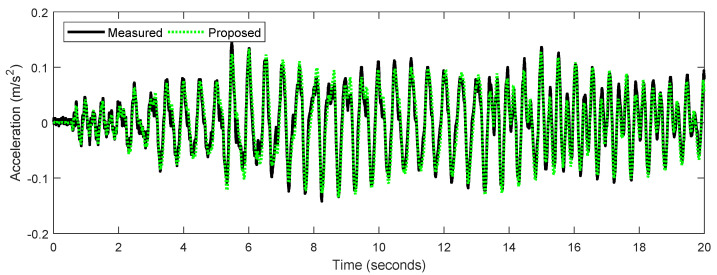
Comparison of middle floor acceleration response from the accelerometer and the proposed method at 2 Hz.

The matched keypoints shown in Figure 7(a-iii) are much more distinct and have greater clarity compared to the keypoints observed in Figure 7(a-i). This is because several unmatched points are removed after implementing the two-step outlier removal process. The Hamming distance-based outlier removal is implemented based on the similarity between each pair of matched keypoints. By comparing the green lines in red circles in Figure 7(b-i,b-ii), it shows that Hamming distance removes motion trajectories that are not similar, whereas IQR score-based outlier removal method focuses on removing the motion vectors based on the direction and length (Equation (4)), so several vertical green lines in the red circle of Figure 7(b-i) are still shown in Figure 7(b-ii), but they are removed from Figure 7(b-iii). As seen in Figure 8, the response obtained from the proposed method matches closely with the measured response.

Next, the accuracy performance of the proposed method is evaluated using root mean square error (RMSE). RMSE is a widely accepted evaluation metric in the performance assessment of computer-vision-based vibration monitoring methods. It measures how far the numerical results are around the observed data and is given by Equation (7) [57].

(7) $$\begin{array}{c} \begin{array}{cc} r & =a_{i}-\hat{a_{i}}\\ \mathrm{RMSE} & ={\left[\frac{1}{N}\sum_{i=1}^{N}r^{2}\right]}^{1/2} \end{array} \end{array}$$

where $\hat{a_{i}}$ is the observed or measured acceleration data captured by the accelerometers mounted on each floor, $a_{i}$ is the acceleration data calculated using vision-based methods, *r* is the residual between the measured data and calculated results, and *N* is the sampling size.

### 6.2. Comparison with Existing Sparse Optical Flow Tracking Methods

The accuracy performance of the proposed method is compared with five existing sparse-optical-flow-based target tracking methods in vibration monitoring. The RMSE and the corresponding error percentages are illustrated in Table 1. The existing methods are combinations of different keypoint detectors (e.g., Shi–Tomasi corner, Harris corner, SURF), LK, and different outlier removals (e.g., MLESAC, bidirectional error). The multi-level optical flow strategy is implemented by combining the SURF detector with LK algorithm [15] and the multi-level LK (MLK) algorithm. It is observed that when the bottom floor is excited with a frequency of 5 Hz and the middle floor with a frequency of 10 Hz, the maximum amplitudes are only around 2 mm and 1 mm, respectively. Hence, the achieved accuracies of all the methods are similar. However, for other cases with larger maximum amplitudes, the proposed method has better accuracy compared to the existing methods.

### 6.3. Comparison with Existing Feature-Matching-Based Tracking Methods

**Figure 9:**
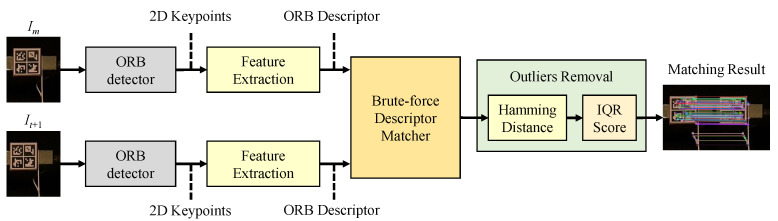
Flowchart of the modified feature-matching-based target tracking method.

In this study, the existing feature-matching-based tracking methods are modified in order to compare with the proposed method which is based on sparse optical flow tracking. Both these target tracking techniques use a set of sparse keypoints. As shown in Figure 9, the modified feature-matching-based method takes the ROIs cropped from the reference, $I_{m}$, and current frames, $I_{t+1}$, as an input. It outputs the matched keypoint pairs that are connected by the colored lines. More specifically, the ORB detector and outlier removal in this method are the same as those used in the proposed sparse-optical-flow-based target tracking method. After applying ORB, each keypoint is described by a 256-bit long binary data string. Then, a brute-force descriptor matcher [54] is employed to estimate the motion vector for each keypoint detected in $I_{m}$.

For feature-matching-based target tracking, a set of keypoints are detected in each video frame independently, so feature-matching-based target tracking can be combined with both fixed-frame and updated-frame strategies. In this comparative study, the modified feature-matching-based method is employed with both frame tracking strategies to calculate the acceleration time histories for each floor of the three-story shear building structure. The feature-matching-based target tracking is implemented in C++ programming language and has three key steps: (i) brute-force matching, (ii) Hamming distance-based outlier removal method, and (iii) IQR score-based outlier removal method.

**Figure 10:**
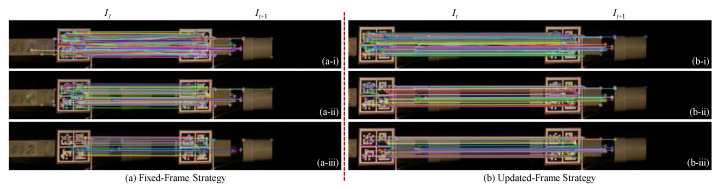
Qualitative example of the feature-matching-based target tracking with (**a**) fixed-frame strategy and (**b**) updated-frame strategy.

Images in Figure 10a show the results calculated by fixed-frame strategy. In accordance with Figure 4, the fixed-frame strategy uses the first frame, $I_{1}$, as the *reference frame* at all times to calculate the displacements. Images in Figure 10b show the results obtained by using the updated-frame strategy. The updated-frame strategy does not use a fixed frame of reference but rather updates it at each time step, which considers two consecutive frames at any time instance such that the previous frame, $I_{t}$, is used as the *reference frame*. Figure 10i shows pairs of initial matched keypoints in the ROIs of *reference frame* and current frame, $I_{t+1}$, after implementing the brute-force matching. The colored lines connect the matched keypoints in the ROI of $I_{1}$ and their corresponding keypoints, 2D position: $\left(x_{i}+\Delta x_{i},y_{i}+\Delta y_{i}\right)$ in $I_{t+1}$. Figure 10ii,iii show the pairs of matched keypoints in the ROIs of *reference frame* and $I_{t+1}$ using colored lines, respectively.

The colored lines shown in Figure 10iii are much more distinct and have greater clarity compared to the lines observed in Figure 10i. This is similar to what we observe in the proposed method as both these target tracking methods employ the same techniques for keypoint detection and outlier removal. However, the matched keypoints using the proposed method shown in Figure 7a are much denser than those obtained using the feature-matching-based method shown in Figure 10b, indicating that the MLK algorithm can find many more matched keypoints than the brute-force method.

Next, the accuracy performance of the proposed method is compared with the modified feature-matching-based target tracking methods. As shown in Table 2, the RMSE and the corresponding error percentages are the least for the proposed method.

### 6.4. Comparison with Existing Dense-Optical-Flow-Based Target Tracking Methods

In this study, a deep-learning-based dense optical flow algorithm [58] is selected as the target tracking method for structural vibration monitoring. This algorithm has the best performance compared to other existing dense optical flow methods and has the ability to handle large displacements with the help of a global motion aggregation module. In contrast to sparse optical flow and feature-matching techniques which explore matched keypoints, dense optical flow is based on a close examination of an ROI. It takes the ROIs of the previous frame, $I_{t}$, and the current frame, $I_{t+1}$, as inputs, and outputs the optical flow of each pixel within ROI. In addition, there is no additional step of outlier removal in a dense optical flow technique. In the current study, the same procedure in Jinag et al. [58] is implemented to train and validate the model. Finally, the pixel value of the center of the generated optical flow map is selected as the relative displacement for the vibrating structure between the current and reference frames. To monitor the structural vibration, the dense-optical-flow-based target tracking method is combined with the updated-frame strategy to calculate the acceleration time histories for each floor of the three-story shear building structure. This tracking method is implemented in the Python programming language, and GPU and PyTorch are employed to speed up the computation.

**Figure 11:**
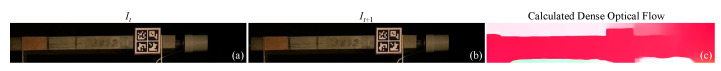
Qualitative example of the target tracking results based on dense optical flow technique.

Figure 11 shows a qualitative result after implementing the dense optical flow algorithm on two consecutive frames. Specifically, Figure 11a,b show the ROIs of $I_{t}$ and $I_{t+1}$, respectively. Figure 11c is the dense optical flow between $I_{t}$ and $I_{t+1}$, which shows the flow vectors of the entire ROI (all pixels) of $I_{t}$. The red color region represents that the object was displaced towards the right, the green color region represents that the object was displaced towards the left, and the white color region represents that the object was not displaced. The pixels with more intensity represent that the object was displaced more. As shown in Figure 11a,b, the top floor was displaced towards the right from $I_{t}$ to $I_{t+1}$, which has the same moving direction (red region) shown in Figure 11c. For these red areas, some of the areas that overlapped with the vibrating structure display the same pixel intensity, which means that these areas of vibration on the building model have the same motion.

Next, the accuracy performance of the proposed method is compared with the dense-optical-flow-based target tracking methods. As shown in Table 3, for harmonic excitations of 2 Hz and 5 Hz, the accuracy of the proposed method is better than the dense-optical-flow-based target tracking method. When the building structure is subjected to harmonic excitation at 10 Hz, their accuracy is almost the same.

### 6.5. Comparison with Existing Template-Matching-Based Target Tracking Methods

In this study, the existing methods [3,9] with ArUco marker as the predefined template are implemented to track the motion of a vibrating structure. ArUco is a system that contains a set of predesigned markers and an algorithm to perform its detection [59]. It is one of the most evolved tools for fiducial marker detection and has been widely used in computer vision applications such as robot navigation and augmented reality. OpenCV [54] is used for automated ArUco marker detection. As shown in Figure 6a, the three ArUco boards were placed on each floor of the structure independently. Compared to existing studies [3,9] which use only a single marker, an ArUco board is designed consisting of four ArUco markers (see Figure 6b) to improve the stability. Specifically, a marker board has a size of 31.8 mm × 31.8 mm and contains four ArUco markers that are 15.4 mm × 15.4 mm. Each marker is composed of a wide black border and an inner binary matrix (high-contrast pattern) which determines their unique ids. As shown in Figure 6d, after applying the ArUco marker detection algorithm for each frame, (x,y)-coordinate and id of each detected ArUco marker are returned, which demonstrates that the structural vibration for each floor is monitored independently even though each floor looks similar. Compared to the first frame, $I_{1}$, of the video, the relative displacement of each detected marker of the current frame, $I_{t+1}$, is calculated using Equation (8).

(8) $$\begin{array}{c} \begin{array}{cc} d_{i_{t+1}} & =\sqrt{{\left(dx_{i_{t+1}}\right)}^{2}+{\left(dy_{i_{t+1}}\right)}^{2}}\\ dx_{i_{t+1}} & =x_{i_{t+1}}-x_{i_{1}}\\ dy_{i_{t+1}} & =y_{i_{t+1}}-y_{i_{1}} \end{array} \end{array}$$

where $d_{i_{t+1}}$ is displacement of marker with id of *i*, $dx_{i_{t+1}}$ and $dy_{i_{t+1}}$ represent displacement in the x-direction and y-direction (see Figure 6), respectively; $\left(x_{i_{1}},y_{i_{1}}\right)$ is the coordinate of the detected marker with id of *i* in $I_{1}$, while $(x_{i_{t+1}}$, $y_{i_{t+1}})$ is the coordinate of the detected marker in $I_{t+1}$. The tracking output for each floor is the average displacement of detected markers, which is calculated as follows:

(9) $$D=\left(\sum_{i=1}^{N}d_{m_{i}}\right)/N$$

where *D* is the average displacement for each floor, $m_{i}$ is the id of detected markers, $d_{m_{i}}$ is the calculated displacement for each marker, and *N* is the number of detected markers.

To monitor the structural vibration, the template-matching-based target tracking method is combined with two frame tracking strategies and is implemented in the C++ programming language. Both template-matching-based and feature-matching-based target tracking techniques detect and recognize targets on each frame, and search matched pairs of targets between the reference and current frames. The only difference is that the template-matching-based target tracking employs ArUco markers as targets, which are physical markers and have been predefined, whereas feature-matching-based target tracking employs keypoints as targets, which are virtual markers and are related to the type of keypoints detector. As shown in Figure 6d, all predefined ArUco markers in the current frame are detected, and then labeled by outer square boxes (white boxes) with unique identified marker numbers (e.g., id=6, white text), which are used to match the detected predefined ArUco markers in the reference frame.

As seen in Table 4, the proposed method performs better than the existing template-matching-based target tracking methods. As mentioned before, predefined templates are easily occluded by adverse factors such as shape deformation and rotation, which can negatively impact the accuracy of template-matching-based target tracking in vibration monitoring.

## 7. Discussion of Results

This section discusses the effect of various components such as ROI selection, the type of outlier removal method, excitation frequency, frame rate, frame strategy, and keypoints tracking techniques on the accuracy of the proposed method.

### 7.1. Effect of ROI Selection and Outlier Removal Methods

When images are processed using vision-based target tracking methods, only the image data within ROI are processed [22]. ROI is defined on the assumption that all keypoints are detected on a rigid structure and they have the same displacement. In vision-based monitoring, the keypoints are regarded as unreliable and removed as outliers if they are either unmatched pairs during target tracking or they are detected in the unreliable regions, such as the regions that are not in motion (e.g., black background in Figure 6), and the regions that experience slight motion relative to the rigid structure (e.g., vertical columns of the three-story shear building structure) [13]. These types of unreliable keypoints can be removed by implementing outlier removal techniques based on methods such as MLESAC [16,17] and bidirectional error detection [18]; however, their performance highly depends on the selection of ROI.

For example, these methods can produce wrong estimates when the number of keypoints detected in the object of interest is not significantly greater than that in any other objects in ROI, which means that the inliers are heavily influenced by the keypoints detected in unreliable regions. The MLESAC algorithm requires an inlier ratio to generate the prior probability, where the inlier ratio should be large enough to ensure the convergence of the maximum likelihood [60], which can be adjusted based on the ROI box. In the bidirectional error detection strategy, the error is defined as the difference between the forward and backward trajectories of a pair of initially matched keypoints [18]. This error is utilized to remove the unmatched pairs of keypoints in terms of similarity, rather than the keypoints detected in unreliable regions or those that have atypical motion directions. In this study, the proposed two-step outlier removal method based on Hamming distance and IQR score outperforms the MLESAC and the bidirectional error detection methods because it considers both the similarity of keypoints as well as their relative motion simultaneously. Even if the ROI contains a large number of unreliable keypoints, the IQR score-based outlier removal methodology performs well as long as the value of constant *r* is properly investigated (Equation (4)). If *r* was set to a high value such as 0.9, a large number of keypoints were removed as outliers which resulted in very few final matched keypoints.

To evaluate the performance of the proposed outlier removal, an additional comparative study is conducted. Specifically, the proposed two-step outlier removal methods are replaced with MLESAC-based and bidirectional error-based outlier removals. As shown in Table 5, the proposed method has an error of less than 3% for all the cases. These results are calculated based on ROIs that have a large number of keypoints detected on the rigid girder and a few keypoints detected on the other components of the experimental setup. During the experiments conducted as a part of this study, the ROIs are selected again to significantly increase the ratio between the number of unreliable keypoints and reliable keypoints.

### 7.2. Effect of Excitation Frequency on the Accuracy of Vision-Based Methods

The RMSE results shown in Table 1, Table 2, Table 3 and Table 4 have smaller values at lower excitation frequencies (e.g., 2 Hz) than those obtained at higher excitation frequencies (e.g., 10 Hz). This occurs because a large number of samples are acquired for lower excitation frequencies compared to higher excitation frequencies within each minima and maxima value of the amplitude of vibration. When the excitation frequency of vibrations is low, the structure undergoes slower oscillations and, hence, the sensors can capture response with a higher resolution, as long as the sampling rate is kept fixed. For example, for a fixed-frame sampling rate of 120 Hz, 30 samples can be acquired between the minima and maxima at 2 Hz excitation frequency, whereas only 6 samples can be collected at 10 Hz excitation frequency. More sampling points between the minima and maxima will result in points that are closer to the minima and maxima. Therefore, a lower RMSE is achieved with a larger number of samples at low excitation frequencies.

### 7.3. Effect of Frame Rate and Frame Strategy

An inaccurate frame rate can cause the calculated acceleration response to deviate from the measured data along the time axis [17]. The frame rate in consumer-grade cameras can be inaccurate and unreliable. For example, the frame rate provided in the camera specifications document is 120 fps, whereas the actual frame rate measured in the metadata was 119.88 fps. The experiment conducted in this study shows that the actual frame rate adopted by the proposed vision-based vibration monitoring can eliminate the drift caused due to inaccurate frame rates and reduce the error in the prediction of acceleration time history. More details can be found in the Supplementary Document.

Furthermore, the technique used to track the frame as a part of the vision-based vibration monitoring methodology can impact the calculated displacement time histories. As shown in Figure 4, for the fixed-frame strategy, every directly calculated absolute displacement is independent of the previously calculated value. Thus, the error does not accumulate at any particular instant of time. In contrast, for the updated-frame strategy, as the displacement at any point of time is dependent on its previous neighbor, the error in the absolute displacement at the current instant will be accumulated subsequently. This causes a drift in the displacement time history and a gradual loss of accuracy in the calculated displacement amplitude.

**Figure 12:**
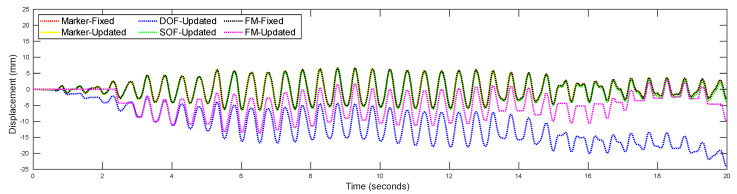
Comparison of vision-based displacement calculation using different frame strategies.

To demonstrate this phenomenon, fixed-frame and updated-frame strategies are utilized and compared as part of the vision-based vibration monitoring methods. Figure 12 illustrates the calculated displacement time history of the middle floor when the three-story shear building structure is subjected to an excitation frequency of 2 Hz. The displacement time histories calculated by using the updated-frame strategy (marker-updated, FM-updated, DOF-updated, and SOF-updated) show the error accumulation when compared to the results calculated by using the fixed-frame strategy (marker-fixed and FM-fixed). It can be seen that the proposed methodology (SOF-updated) has negligible drift when compared to the existing vibration monitoring methodologies [9,16] that use the updated-frame strategy.

To correct the drift along the amplitude, Hoskere et al. [9] proposed to use the size and shape of a fiducial marker to compensate for the perspective distortions, and Lydon et al. [16] utilized a known stable concrete block location in the frame as an anchor point to correct and compensate for the camera movement. In comparison, although the methodology proposed in this study is not focused on addressing the issue of amplitude drift, it is able to eliminate much of the drift in the calculated displacements without implementing any specific algorithm or device as a correction technique.

### 7.4. Effect of Sparse Optical Flow versus Feature-Matching Technique on Keypoints Tracking

Both feature-matching and sparse-optical-flow-based structural vibration monitoring techniques are implemented by tracking the keypoints on the vibrating structure, but the number of errors obtained by implementing the sparse-optical-flow-based method are fewer than those obtained from the feature-matching-based method (see Table 2).

Feature-matching-based target tracking detects the keypoints of two frames independently, and then searches for matched keypoints in different frames by matching similar descriptors. Although numerous keypoint detectors have been developed, it is still difficult for one keypoint detector to consider all factors such as viewpoint, illumination, scale, blur, and compression, which affects the accuracy of keypoint detection [61]. For the displacement time history obtained by feature-matching-based target tracking with updated-frame strategy (see Figure 12), several peaks near the beginning and the end of the magenta curve are flat. This means that no motion of vibration is detected between two continuous frames.

In contrast, the proposed sparse-optical-flow-based target tracking detects keypoints in the reference frame, and then searches for matched keypoints in the current frame by estimating the motion vector of each keypoint based on the LK algorithm. The LK algorithm [11] searches for matched points based on pixel intensity, rather than the similarity of descriptors. Moreover, ORB utilizes FAST as the feature detector due to its advantage over issues such as noise, blur, and compression, because the scale space and denoising are not considered [61]. Therefore, the proposed sparse-optical-flow-based technique outperforms the feature-matching-based technique for tracking various keypoints in structural vibration monitoring.

## 8. Summary and Conclusions

This research proposes a new method for computer-vision-based structural vibration monitoring. Traditionally, vibration monitoring of structural systems can be achieved by installing discrete sensors, such as accelerometers, to acquire the motion response of the structure and by utilizing data acquisition systems such as oscilloscopes to collect the data from sensors. However, such traditional measurement techniques have several disadvantages, such as the expensive installation and subsequent maintenance of sensors. To overcome these limitations, computer-vision-based methods can be employed, where a camera records the movement of the structure to detect certain target keypoints, and a target tracking algorithm is designed to obtain the structural motions such as the acceleration time history. The method proposed in this study is validated by comparing the vision-based acceleration results with those obtained from accelerometers on a three-story shear building structure in the laboratory. The accuracy of the proposed method is also compared with existing computer-vision-based tracking techniques. It is observed that the proposed target tracking method achieved the highest accuracy for vibration monitoring of the structure in the experimental setup. The effect of various components used as a part of the proposed methodology are investigated and described, such as the selection of ROI and outlier removal methodologies on the accuracy of matched keypoints, determination of the frame rate used by the acquisition camera on time drift, and implementation of fixed frame versus updated frame on amplitude drift. The key conclusions of the study are summarized as follows:

1. A sparse-optical-flow-based target tracking is enhanced by the use of various components such as the ORB keypoint detector, multi-level optical flow algorithm, and outlier removal techniques. Existing sparse-optical-flow-based computer vision methods are known to have disadvantages such as tracking large displacements. This limitation is improved by the use of two outlier removal methods and multi-point movement tracking to obtain a comprehensive assessment of the structural response. The comparison results illustrated in Table 1 show that the proposed method exhibits higher accuracy than existing methods for cases with larger displacement amplitudes and similar accuracy for all other cases.
2. Validation of the proposed vision-based target tracking method is performed with a shear building experimental setup. The structure is subjected to transient vibrations at three excitation frequencies with varying amplitudes. Figure 8 illustrates the calculated versus measured acceleration time history. It is observed that the target tracking method is able to detect the structural motion and calculate its acceleration at numerous points of the structure with great accuracy.
3. Various other computer-vision-based methods, such as dense-optical-flow-based, feature-matching-based, and template-matching-based target tracking, are compared with the proposed methodology to check for its accuracy. The limitations of existing methodologies and the proposed enhancements are summarized as follows:
  - Template-matching-based target tracking approaches have an inherent disadvantage due to adverse factors, such as partial occlusion, shape deformation and rotation, which can affect the detection of predefined templates. Therefore, the proposed sparse-optical-flow-based method attempts to track various keypoints on the vibrating structure without the use of external templates. As shown in Table 4, it is observed that the proposed method achieves higher accuracies than the existing template-matching-based target tracking approaches.
  - Another similar keypoint tracking approach, called the feature-matching-based target tracking method, is also compared. However, the keypoint detectors implemented as a part of this existing method have some disadvantages, such as the illumination, scale, blur, and compression of images captured during structural vibrations. In the proposed sparse-optical-flow-based method, these limitations are corrected by the use of the ORB FAST keypoint detector in combination with the LK algorithm to detect a higher number of matched keypoints (Table 2).
  - Additionally, Table 3 shows that the proposed method is observed to perform quite similarly to the existing dense-optical-flow-based technique which compares predefined ROI templates without any outlier removal approach. However, for lower excitation frequencies, the computer-vision-based technique proposed in this study with outlier removal outperforms the existing dense-optical-flow-based method.

## Figures and Tables

**Table 1:** RMSE (mm) and its error percentages (%) for sparse optical flow tracking methods.

Freq	Methods	Bottom (%)	Middle (%)	Top (%)
2 Hz	Shi–Tomasi corner + LK [10,14]	0.0184 (+5.747)	0.0170 (+11.842)	0.0216 (+4.854)
	Harris corner + LK [9]	0.0181 (+4.023)	0.0170 (+11.842)	0.0212 (+2.913)
	SURF + LK [15]	0.0253 (+45.402)	0.0165 (+8.553)	0.0226 (+9.709)
	SURF + LK + MLESAC [16]	0.0178 (+2.299)	0.0166 (+9.211)	0.0226 (+9.709)
	SURF + LK + Bidir. error [18]	0.0177 (+1.724)	0.0166 (+9.211)	0.0224 (+8.738)
	SURF + MLK	0.0321 (+84.483)	0.0162(+6.579)	0.0213 (+3.398)
	Proposed	0.0174 (+0)	0.0152 (+0)	0.0206 (+0)
5 Hz	Shi–Tomasi corner + LK [10,14]	0.0217 (+1.878)	0.1225 (+151.540)	0.2668 (+352.971)
	Harris corner + LK [9]	0.0218 (+2.347)	0.1267 (+160.164)	0.2851 (+384.041)
	SURF + LK [15]	0.0272 (+27.700)	0.1307 (+168.378)	0.2815 (+377.929)
	SURF + LK + MLESAC [16]	0.0222 (+4.225)	0.1338 (+174.743)	0.2799 (+375.212)
	SURF + LK + Bidir. error [18]	0.0223 (+4.695)	0.1320 (+171.047)	0.2740 (+365.195)
	SURF + MLK	0.0264 (+23.944)	0.1246 (+155.852)	0.1492 (+153.311)
	Proposed	0.0213 (+0)	0.0487 (+0)	0.0589 (+0)
10 Hz	Shi–Tomasi corner + LK [10,14]	0.5975 (+150.945)	0.0639 (+0.157)	0.3693 (+122.336)
	Harris corner + LK [9]	0.6058 (+154.431)	0.0638 (+0)	0.3731 (+124.624)
	SURF + LK [15]	0.5944 (+149.643)	0.0692 (+8.464)	0.4062 (+144.551)
	SURF + LK + MLESAC [16]	0.6112 (+156.699)	0.0686 (+7.524)	0.4113 (+147.622)
	SURF + LK + Bidir. error [18]	0.6070 (+154.935)	0.0687 (+7.680)	0.4097 (+146.659)
	SURF + MLK	0.2830 (+18.858)	0.0661 (+3.605)	0.1886 (+13.546)
	Proposed	0.2381 (+0)	0.0647 (+1.411)	0.1661 (+0)

Freq: frequencies of harmonic excitations; red value: lowest value, i.e., BEST accuracy.

**Table 2:** RMSE (mm) and its error percentages (%) for feature-matching-based tracking methods.

Freq.	Method	Bottom (%)	Middle (%)	Top (%)
2 Hz	FM-Fixed	0.0243 (+39.655)	0.0235 (+54.605)	0.0266 (+29.126)
	FM-Updated	0.0407 (+133.908)	0.0349 (+129.605)	0.0366 (+77.670)
	Proposed	0.0174 (+0)	0.0152 (+0)	0.0206 (+0)
5 Hz	FM-Fixed	0.0839 (+293.897)	0.0862 (+77.002)	0.0950 (+61.290)
	FM-Updated	0.0725 (+240.376)	0.0798 (+63.860)	0.0813 (+38.031)
	Proposed	0.0213 (+0)	0.0487 (+0)	0.0589 (+0)
10 Hz	FM-Fixed	0.2499 (+4.956)	0.1071 (+65.533)	0.1841 (+10.837)
	FM-Updated	0.2441 (+2.520)	0.0977 (+51.005)	0.1759 (+5.900)
	Proposed	0.2381 (+0)	0.0647 (0)	0.1661 (+0)

Freq: frequencies of harmonic excitations; red value: lowest value, i.e., BEST accuracy.

**Table 3:** RMSE (mm) and error percentages (%) for dense optical flow tracking methods.

Freq.	Method	Bottom (%)	Middle (%)	Top (%)
2 Hz	DOF-Updated	0.0178 (+2.299)	0.0173 (+13.816)	0.0221 (+7.282)
	Proposed	0.0174 (+0)	0.0152 (+0)	0.0206 (+0)
5 Hz	DOF-Updated	0.0247 (+15.962)	0.0582 (+19.507)	0.0619 (+5.093)
	Proposed	0.0213 (+0)	0.0487 (+0)	0.0589 (+0)
10 Hz	DOF-Updated	0.2382 (+0.042)	0.0637 (+0)	0.1670 (+0.542)
	Proposed	0.2381 (+0)	0.0647 (+1.570)	0.1661 (+0)

Freq: frequencies of harmonic excitations; red value: lowest value, i.e., BEST accuracy.

**Table 4:** RMSE (mm) and error percentages (%) of existing template-matching-based target tracking methods.

Freq.	Method	Bottom (%)	Middle (%)	Top (%)
2 Hz	Marker-Fixed	0.0186 (+6.897)	0.0173 (+13.816)	0.0219 (+6.311)
	Marker-Updated	0.0341 (+95.977)	0.0414 (+172.368)	0.0245 (+18.932)
	Proposed	0.0174 (+0)	0.0152 (+0)	0.0206 (+0)
5 Hz	Marker-Fixed	0.0357 (+67.606)	0.0583 (+19.713)	0.0699 (+18.676)
	Marker-Updated	0.0512 (+140.376)	0.1938 (+297.947)	0.1472 (+149.915)
	Proposed	0.0213 (+0)	0.0487 (+0)	0.0589 (+0)
10 Hz	Marker-Fixed	0.2391 (+0.420)	0.0707 (+9.274)	0.1704 (+2.589)
	Marker-Updated	0.6249 (+162.453)	0.1367 (+111.283)	0.1697 (+2.167)
	Proposed	0.2381 (+0)	0.0647 (+1.570)	0.1661 (+0)

Freq: frequencies of harmonic excitations; red value: lowest value, i.e., BEST accuracy.

**Table 5:** RMSE (mm) and its error percentages (%) for different outliers.

Methods	Freq	Bottom (%)	Middle (%)	Top (%)	ROI Size (Pixels)	Image Processing Speed (fps)
ORB + MLK + MLESAC	2 Hz	0.0177 (+2.299)	***0.0152 (0)***	***0.0204 (0)***	318×1006	***17.13***
	5 Hz	0.0211 (+1.442)	0.0721 (+48.049)	0.0608 (+3.932)		
	10 Hz	0.2477 (+4.032)	0.0740 (+16.352)	0.1685 (+1.445)		
ORB + MLK + Bidir. error	2 Hz	***0.0173 (0)***	***0.0152 (0)***	***0.0204 (0)***	318×1006	8.89
	5 Hz	***0.0208 (0)***	0.0656 (+34.702)	***0.0585 (0)***		
	10 Hz	0.2476 (+3.990)	***0.0636 (0)***	0.1681 (+1.204)		
Proposed	2 Hz	0.0174 (+0.578)	***0.0152 (0)***	0.0206 (+0.980)	318×1006	13.77
	5 Hz	0.0213 (+2.404)	***0.0487 (0)***	0.0589 (+0.684)		
	10 Hz	***0.2381 (0)***	0.0647 (+1.700)	***0.1661 (0)***		

Green, cyan, and orange values represent the results of the vibration of each floor under excitation frequencies with 2 Hz, 5 Hz, and 10 Hz, respectively. ***Bold text***: the lowest value of each case, i.e., BEST performance.

## Data Availability

Some or all data, models, or codes that support the findings of this study are available from the corresponding author upon reasonable request.

## Supplementary Materials

The following are available online at https://www.mdpi.com/article/10.3390/s22186869/s1, Table S1. Technical Specifications of Excitation System. Table S2. Technical Specifications of Reference System. Table S3. Camera Settings. Table S4. Calculated Distortion Related Parameters. Figure S1. Example of camera calibration. (a) image before distortion removal; (b) one of the snapshots that is used for camera calibration; (c) image after distortion removal. Figure S2. Strategy that is used to create videos with different numbers of frames. Figure S3. Evaluation of accuracy of the calculated accelerations for different actual frame rates of the video. The video captures the vibration of the bottom floor of the structure under the excitation frequency of 10 Hz. Dense optical flow algorithm with updated-frame strategy (DOF) is used to calculate the accelerations.
