# Supplementary material for: Computer-Vision-Based Vibration Tracking Using a Digital Camera: A Sparse-Optical-Flow-Based Target Tracking Method
Source: Sensors (Basel). 2022 Sep 11;22(18):6869. doi: 10.3390/s22186869 (PMC9504661; doi:10.3390/s22186869)
Supplement: Supplementary file 1 [file sensors-22-06869-s001.zip › sensors-1873325-supplementary.pdf]

sensors

MDPI

Article

Computer-Vision-Based Vibration Tracking Using a Digital Camera: A Sparse-Optical-Flow-Based Target Tracking Method

Guang-Yu Nie, Saran Srikanth Bodda, Harleen Kaur Sandhu, Kevin Han * and Abhinav Gupta

Department of Civil, Construction, and Environmental Engineering, North Carolina State University, Raleigh, NC 27695, USA
* Correspondence: kevin_han@ncsu.edu

Citation: Nie, G.-Y.; Bodda, S.S.; Sandhu, H.K.; Han, K.; Gupta, A. Computer-Vision-Based Vibration Tracking Using a Digital Camera: A Sparse-Optical-Flow-Based Target Tracking Method. *Sensors* **2022**, *22*, 6869. https://doi.org/10.3390/s22186869

Academic Editors: Alexey Pak, Steffen Reichel and Jan Burke

Received: 2 August 2022
Accepted: 7 September 2022
Published: 11 September 2022

Publisher's Note: MDPI stays neutral with regard to jurisdictional claims in published maps and institutional affiliations.

Copyright: © 2022 by the authors. Licensee MDPI, Basel, Switzerland. This article is an open access article distributed under the terms and conditions of the Creative Commons Attribution (CC BY) license (https://creativecommons.org/licenses/by/4.0/).

1. Experimental Devices

The technical specifications of instruments used in the **Excitation System**, **Reference System**, and **Camera** are tabulated in Table S1, Table S2, and Table S3, respectively.

Table S1. Technical Specifications of Excitation System

**Component**	**Company/Model**	**Technical Specifications**
Waveform Generator	TEGAM/Model 2732	Bandwidth: $1\mu Hz$ to $50MHz$<br>2 Channels
Digital Power Amplifier	Crown/Model XTI 2000	Frequency Response: $\pm 0.25dB$ from $20Hz$ to $20kHz$ at $1watt$ into $4ohms$<br>Voltage Gain at $1kHz$, $8ohm$ rated output: $32.9dB$<br>Signal to Noise Ratio (below rated $1kHz$ power at $8ohms$): $105dB$ (A weighted)
Electromagnetic actuator	Vibration Test Systems /Model VTS-600	Single Vibrator System<br>Rated Force, peak sine wave: $600lbf$<br>Useful Frequency Range: 2 to $5000Hz$<br>Maximum Acceleration, bare table: $140g$<br>Rated Velocity: $70in/sec$
Shake Table	-/-	Material: aluminum<br>Size($w \times h \times l$): $303.0mm \times 12.5mm \times 606.3mm$

Table S2. Technical Specifications of Reference System

**Component**	**Company/Model**	**Technical Specifications**
Accelerometer	PCB Piezotronics/Model 308B02	Sensitivity (mV/g): 1000<br>Frequency Range ($\pm$10%): 250-3000<br>Temp Range (°F): -65/250<br>Mass ($grams$): 65<br>Single Axis
Sensor Signal Conditioner	PCB Piezotronics/Model 482C05	4 Channels<br>Sensor Input Type: ICP®
Oscilloscope	Tektronix/MDO34 3-BW-350	4 Analog Channels<br>16 Digital Channels<br>Bandwidth (MHz): 350<br>Sample Rate (GS/s): 2.5<br>Record Length ($MPts$): 10
Software	-/TekScope Utility	Outputs: .CSV file

Table S3. Camera Settings

Item	Value	Units	Item	Value	Units
Frame Rate	120	Hz	Resolution	1280 x 960	$Pixels(p)$
Aperture	F9	N/A	Shutter Speed	1/8000	$Seconds(s)$
ISO	800	1	Focus Mode	AF-S	N/A

2. Video Image Distortion Removal for Vision-Based Sensing System

This section describes the method to remove the distortion of video images captured by the cameras used in this study.

2.1. Video Image Distortion Removal

Figure S1 (a) shows an image directly captured by the camera, where a 9 × 6 classical black-white chessboard pattern is used as the calibration board and placed in front of the three-story structure. The distance between two yellow corner points as shown in Figure S1 (a) is 180 mm. Then, twenty snapshots of the chessboard are taken by the camera from different angular views. Figure S1 (b) shows one of the snapshots, where all corners of the chessboard are labeled by colored circles. Finally, OpenCV takes all snapshots as input and estimates all five camera parameters (i.e., k_1, k_2, k_3, p_1, p_2) that are used to remove the optical distortion of the captured videos. The distortion parameters of the camera used in this study are shown in Table S4, and the arithmetical mean of the errors calculated for all snapshots is 0.1782 pixel. Figure S1 (c) illustrates the result after applying the distortion removal on Figure S1 (a). Two red dots are the calibrated locations at which the two original points (yellow dots in Figure S1 (a)) project after applying the projection transformation matrix.

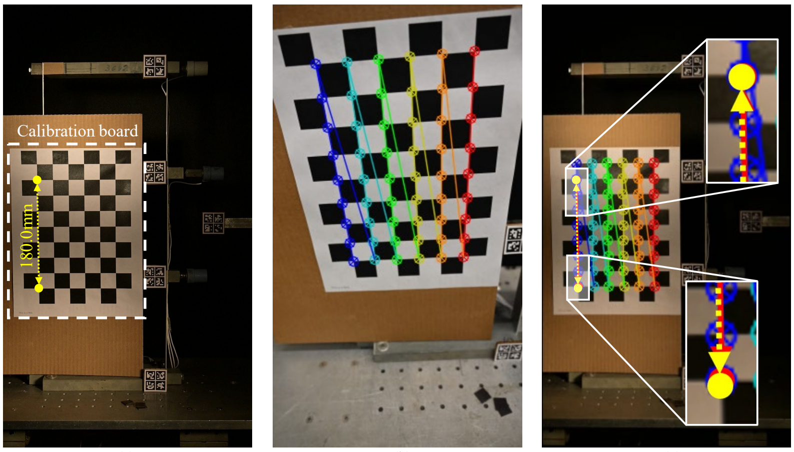

(a) (b) (c)

Figure S1. Example of camera calibration. (a) image before distortion removal; (b) one of the snapshots that is used for camera calibration; (c) image after distortion removal.

Table S4. Calculated Distortion Related Parameters

	k_1	k_2	k_3	p_1	p_2
**Value**	0.00224349	-0.15135992	0.37956948	0.00679483	-0.00144892

2.2. Scale Ratio Calculation

The scale ratio (units: $mm/pixels$) represents a relationship between the actual spatial coordinates and the image pixel coordinates. In this study, the optical axis of the camera is perpendicular to the object motion plane, and then the scale ratio is determined via

a known dimension in the chessboard. As shown in Figure S1 (c), the physical distance between the two red dots on the chessboard is 180 mm, and the scale ratio is:

$$s = 180 \times [(x_1 - x_2)^2 + (y_1 - y_2)^2]^{-1/2} \quad (1)$$

where, (x_1, y_1), (x_2, y_2) are coordinates of the two red dots in the image after distortion removal.

3. Effect of Actual Frame Rate of Camera on Vision-Based Acceleration Calculation

As shown in the bottom sub-figures in supplemental materials, with increasing time, the calculated acceleration deviates from the measured data in the x-direction, where the drift becomes larger as the time increases. Furthermore, the official frame rate of our camera is $120fps$, but the actual frame rate is around $119.88fps$. We designed an experiment to explore whether or not the actual frame rate of the camera has a relationship with the drift in the x-direction. In this experiment, we combine dense optical flow-based motion tracking algorithm and PFT strategy to calculate the acceleration time histories on the videos with various numbers of frames.

Firstly, we videoed the vibration of the bottom floor under the excitation frequency of 10Hz and cut a video segment with 1200 frames. Then, we created three videos with various numbers of frames (i.e., 1198, 1199, 1200). Figure S2 represents a video with 1200 frames (red, blue, and green planes), a video containing 1199 frames was created by removing the 1200th (green plane) frame, so 1199 frames (red and blue planes) are contained in the 10-second video. Another video containing 1198 frames was created by removing the 1st (red plane) and 1200th (green plane) frames, so 1198 frames (blue planes) are contained in the 10-second video.

Figure S3 (a) compares the measured acceleration with the predicted acceleration based on three videos, where the measured data (Measured) captured by the oscilloscope is labeled by the orange line, acceleration time histories calculated by the vision-based method for a video with 1200 frames (DOF-1200pts), and 1199 frames (DOF-1199pts), 1198 frames (DOF-1198pts) are labeled by the blue, red, and green line, respectively. Figure S3 (b) is the error graph for each case, where the errors of the predicted results are also labeled by blue, red, and green line, respectively. As shown in Figure S3 (a), the red line is closer to the orange line than either the blue or green line, and, as shown in Figure S3 (b), the magnitude of the red line is lower than that of the other two lines. We also calculate the RMSE for each video. The RMSE for the videos with 1200, 1199, and 1198 frames is 0.238205 mm, 0.081139 mm, and 0.153262 mm, respectively. According to the results graphs, the error of the video with 1199 frames is lower than that of the videos with 1200 and 1198 frames, while the error of the video with 1198 frames is lower than that of the video with 1120 frames, which means that the smaller the difference between the actual camera frame rate and a video frame rate, the lower the error of the predicted acceleration. In summary, the frame rate of the video which is close to the actual frame rate of the camera will eliminate the drift and decrease the error in the x-direction of the acceleration time history.

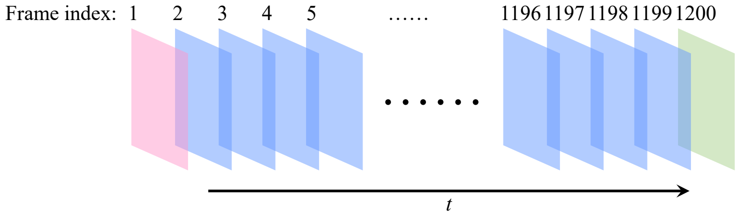

Figure S2. Strategy that is used to create videos with different numbers of frames.

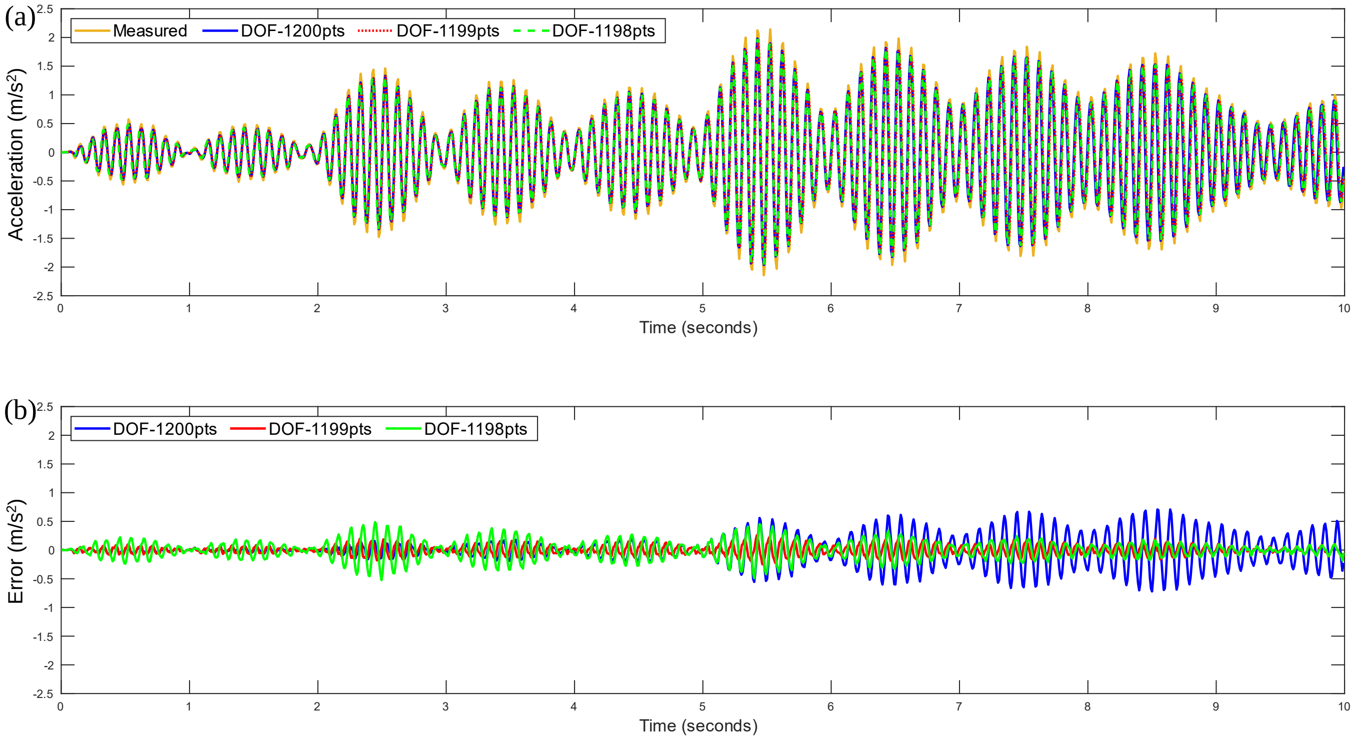

Figure S3. Evaluation of accuracy of the calculated accelerations for different actual frame rates of the video. The video captures the vibration of the bottom floor of the structure under the excitation frequency of 10Hz. Dense optical flow algorithm with updated-frame strategy (DOF) is used to calculate the accelerations. (a) Predicted accelerations; (b) Error/Difference between data calculated by vision-based method and data measured by accelerometer mounted on bottom floor. 1198pts, 1199pts and 1200pts: videos recorded by cameras with actual frame rate of 119.8 Hz, 119.9 Hz and 120.0 Hz, respectively.
